# Compartmentalized Replication of SARS-Cov-2 in Upper vs. Lower Respiratory Tract Assessed by Whole Genome Quasispecies Analysis

**DOI:** 10.3390/microorganisms8091302

**Published:** 2020-08-26

**Authors:** Martina Rueca, Barbara Bartolini, Cesare Ernesto Maria Gruber, Antonio Piralla, Fausto Baldanti, Emanuela Giombini, Francesco Messina, Luisa Marchioni, Giuseppe Ippolito, Antonino Di Caro, Maria Rosaria Capobianchi

**Affiliations:** 1 National Institute for Infectious Diseases, L’Istituto Nazionale per le Malattie Infettive (INMI), “Lazzaro Spallanzani” Istituto di Ricovero e Cura a Carattere Scientifico (IRCCS), 00149 Rome, Italy; martina.rueca@inmi.it (M.R.); cesare.gruber@inmi.it (C.E.M.G.); emanuela.giombini@inmi.it (E.G.); francesco.messina@inmi.it (F.M.); luisa.marchioni@inmi.it (L.M.); giuseppe.ippolito@inmi.it (G.I.); antonino.dicaro@inmi.it (A.D.C.); maria.capobianchi@inmi.it (M.R.C.); 2 Molecular Virology Unit, Microbiology and Virology Department, Fondazione IRCCS Policlinico San Matteo, 27100 Pavia, Italy; a.piralla@smatteo.pv.it (A.P.); fausto.baldanti@unipv.it (F.B.); 3 Department of Clinical-Surgical, Diagnostic and Pediatric Sciences, Università Degli Studi di Pavia, 27100 Pavia, Italy

**Keywords:** coronavirus, variability, SARS-COV-2, COVID-19, quasispecies

## Abstract

We report whole-genome and intra-host variability of SARS-Cov-2 assessed by next generation sequencing (NGS) in upper (URT) and lower respiratory tract (LRT) from COVID-19 patients. The aim was to identify possible tissue-specific patterns and signatures of variant selection for each respiratory compartment. Six patients, admitted to the Intensive Care Unit, were included in the study. Thirteen URT and LRT were analyzed by NGS amplicon-based approach on Ion Torrent Platform. Bioinformatic analysis was performed using both realized in-house and supplied by ThermoFisher programs. Phylogenesis showed clade V clustering of the first patients diagnosed in Italy, and clade G for later strains. The presence of quasispecies was observed, with variants uniformly distributed along the genome and frequency of minority variants spanning from 1% to ~30%. For each patient, the patterns of variants in URT and LRT were profoundly different, indicating compartmentalized virus replication. No clear variant signature and no significant difference in nucleotide diversity between LRT and URT were observed. SARS-CoV-2 presents genetic heterogeneity and quasispecies compartmentalization in URT and LRT. Intra-patient diversity was low. The pattern of minority variants was highly heterogeneous and no specific district signature could be identified, nevertheless, analysis of samples, longitudinally collected in patients, supported quasispecies evolution.

## 1. Introduction

By 26 May, 2020, over five million confirmed cases of Covid-19, people infected with the novel betacoronavirus SARS-CoV-2, were reported worldwide, leading to more than 300,000 deaths [1]. Italy was the first country in Europe to be severely affected. At the beginning of February 2020, an outbreak of infections occurred in the northern Italian regions of Lombardy and Veneto, caused by local transmission, and later on, all the other regions were affected. On March 10, the spread of the outbreak led to the lockdown of the entire country to contrast viral transmission. Infection of SARS-CoV-2 is mainly transmitted by respiratory droplets and causes symptoms such as fever, cough, dyspnea, and respiratory difficulties sometimes resulting in Intensive Care Unit (ICU) admission.

There is much to know about this pandemic, and researchers from all over the world are putting their efforts into studying the evolution, pathogenesis, and transmission of this virus. Virus sequencing data (consensus) have been quickly collected from all over the world and shared through several databases (GISAID, GenBank), but few investigations have been carried out on intra-host variability in the different body compartments and its effects on clinical manifestations.

Here, we report the analysis of intra-host variability in six patients with severe presentation of COVID-19 in two different body districts: upper respiratory tract (URT) vs. lower respiratory tract (LRT).

The overall aim was to establish possible relationships among viral genome heterogeneity and possible compartmentalization of variants in patients with severe disease. Whole-genome assembling as well as the phylogenetic analysis were also performed.

## 2. Materials and Methods

### 2.1. Next Generation Sequencing (NGS) of Clinical Samples

Six patients with COVID-19 severe presentations and admitted to ICU were included in the study (Table 1) and clinical specimens from upper respiratory tract (URT), namely nasopharyngeal swabs (NPS) and lower respiratory tract (LRT), namely bronchoalveolar lavages (BAL), were collected and analyzed. For Pt1, two BAL samples collected at eight days apart were analyzed. All specimens were collected from late January to mid-March. Four patients were hospitalized at the National Institute for Infectious Diseases “L. Spallanzani” (INMI) in Rome, among these the first two cases of COVID-19 in Italy [2]. The remaining two patients were hospitalized at Fondazione IRCCS Policlinico San Matteo in Pavia. Demographic data and epidemiological links are reported in Table 1. In all patients, the presence of SARS-CoV-2 RNA was detected in respiratory specimens by using a real-time reverse transcription-polymerase chain reaction (RT-PCR) assay (Table 1). The sequence investigation of patient samples was approved by the Ethics Committee of INMI (Ethical approval: no. 70/2018(17/12/2018)).

After nucleic acid extraction performed with QiaSymphony automatic extractor using the DPS Virus/Pathogen Midi Kit (QIAGEN, Hilden, Germany); NGS was carried out on an Ion Torrent S5 platform using an Ion AmpliSeq SARS-CoV-2 Panel following the manufacturer’s instructions (ThermoFisher Scientific, Waltham, MA, USA).

### 2.2. Data Analysis

De novo assembly was performed using Trinity v2.8.4 [4]; major contigs were mapped to the Wuhan Hu-1 reference genome and merged with Trinity Geneious 2019.2.3 to reconstruct whole-genome sequences. SARS-CoV-2 sequences posted on GISAID up to 8 May, 2020 [5] were collected. All complete (high coverage) sequences from samples collected in Italy were selected, while the others were clustered at 100% using cd-hit [6]. Multiple sequence alignment was obtained with MAFFT v7.271.

Phylogenetic analysis was performed with IQ-TREE: transition model with empirical base frequencies and invariable sites (TIM + F + I) was selected with ModelFinder; the best tree was found performing 1000 bootstrap ultrafast replicates. Representative sequences were identified using the phylogenetic framework proposed by Rambaut et al. [7], selecting only lineages that were most related to sequences from Italy.

Variants were selected using Thermo Fisher Official Variant Caller (TVC) version 5.12. Parameters were relaxed to include variants up to 1%, and variants with a Phred-like score >30 were considered for this purpose (Table S3). All the results (in VCF format) were then normalized and merged with BCFTOOLS software (Version: 1.10.2) [8].

To calculate the nucleotide diversity, raw reads with mean quality Phred score >20 were selected, trimmed with Trimmomatic v.0.36 [9], and mapped to the reference genome of SARS-CoV-2 (GenBank: MN908947.3) using BWA v.0.7.12 [10]. Mpileup files were generated by Samtools v.1.3.1 [8] and analyzed with a homemade python script (ViVOfinder tool, freely available under https://github.com/cesaregruber/ViVOfinder). The intra-sample variability of the virus genome was evaluated for all BAL and NPS samples: only the positions with a minimum coverage of 20 reads were considered.

Statistical analysis was performed with GraphPad to assess BAL and NPS median diversity using Wilcoxon matched-pairs signed rank test.

## 3. Results

NGS was performed on 13 respiratory samples (six NPS and seven BAL) from six patients (two tourists from China, four Italian residents) obtaining on average 2.0 × 10^6^ reads per sample (range 0.8–4.2 × 10^6^). Mean value and range of coverage for SARS-CoV-2 genome of reads obtained by NGS for each analyzed sample are represented in Table S1.

Consensus sequences are described in Table 2, and differences with the Wuhan-Hu reference genome (GenBank: MN908947) are highlighted. All consensus sequences have been submitted to GISAID and GeneBank.

### 3.1. Phylogenetic Analysis

**Figure 1:**
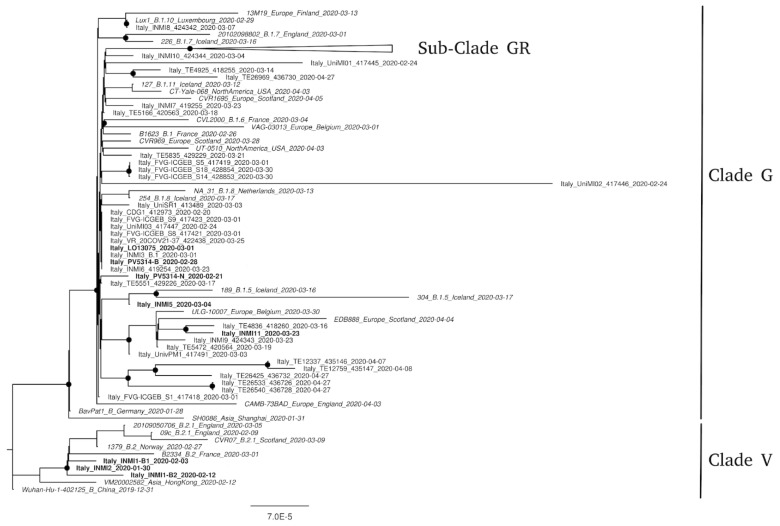
Phylogenetic analysis of SARS-CoV-2 strains circulating in Italy. Available genomes were retrieved from GISAID for 8 May 8, 2020, and all sequences from Italy were selected (see Methods). Representative sequences from other countries, selected to be the most related to sequences from Italy, are included in the tree. Nodes with a bootstrap value of at least 70% are marked with a black dot. Scale bar represents the number of substitutions per site. All not Italians strains are reported in italics; sequences described in this work are reported in bold; sub-clade GR (that includes 41 sequences from Italy) is collapsed.

The phylogenetic tree showed that all sequences obtained from specimens collected in Italy and available on GISAID at the time of this study clustered into two phylogenetic groups. These groups, corresponding to B1 and B2 lineages defined by Rambaut et al. [7], were referred to as G and V clades according to GISAID nomenclature [5]. As previously reported [2,11], only INMI1 and INMI2 were included in V clade, while all other sequences from Italian specimens clustered in G clade. Inside this clade, several clusters with supporting bootstrap value >70% were present. INMI11 fell within one of these, while LO-13077 (Pt6) and PV-5314-B (Pt5) grouped with other sequences from Friuli Venezia Giulia, Veneto, Lombardia, and Lazio. None of the study sequences clustered within the newly described GR subclade [5]. In the majority of patients, the consensus sequences from the different compartments were identical and only one sequence was shown in the phylogenetic tree (Figure 1). In two cases, the consensus sequences from different respiratory compartments (BAL vs. NPS from Pt5–PV-5314) or collected at different times (BAL T1 + NPS T1 vs. BAL T2 from Pt1–INMI1) showed few nucleotide differences along the whole-genome (1 to 3 nt), causing distancing from the corresponding phylogenetic cluster.

### 3.2. Genetic Variability of Whole-Genome Consensus Sequences

On the whole, 15 nt substitutions were observed in the consensus sequences from 13 samples, as compared to the reference genome (Table 2). In detail, one substitution was in the 5′ untranslated region (UTR), while seven synonymous and seven non-synonymous substitutions were across the coding regions, with an average number of 4.4 polymorphic sites observed between samples (2.3 and 2.2 for BAL and NPS, respectively).

Among the variants, the typical clade-specific signatures were observed including C241T in 5′UTR, A23403G leading to D614G (in S protein gene), C14408T leading to P4715L and the synonymous C3037T (in orf1ab) in G clade, and G11083T leading to L2606F (orf1ab) as well as G26144T leading to G251V (orf3a) in V clade.

### 3.3. Diversity and Variation Analysis

**Figure 2:**
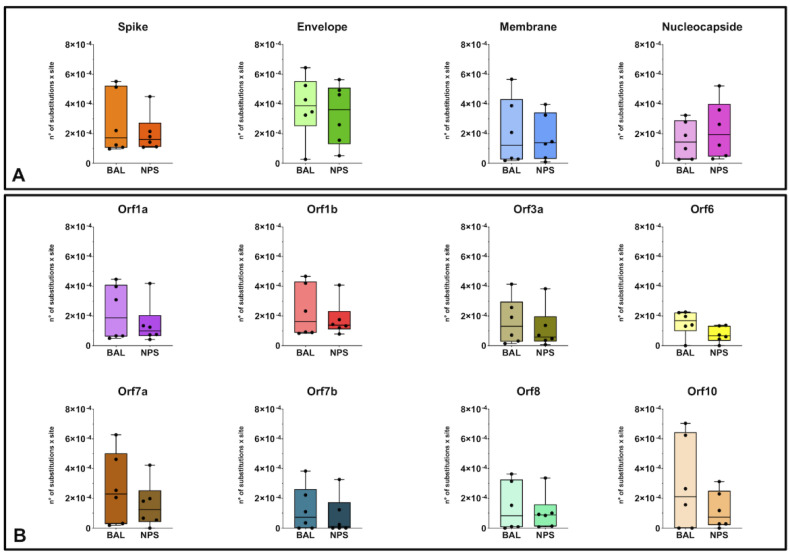
Intra-host nucleotide diversity of SARS-CoV-2 genomic regions in BAL and NPS samples: (**A**) structural proteins regions; (**B**) non-structural proteins regions.

The heterogeneity of SARS-CoV-2 genomes in different respiratory compartments was evaluated by analyzing the sequence variants detected in each single sample. Considering the overall diversity along the whole-genome sequences in BAL and NPS specimens, the median value was 1.92 and 1.24 nt substitution × 10^−4^/site, with no significant differences between URT and LRT (Table S2). More in detail, nucleotide diversity was also calculated for different SARS-CoV-2 genome regions, divided in structural and non-structural proteins (Figure 2, panel A and B). As shown in Figure 2, the sequences showed a certain degree of intrinsic heterogeneity. Comparing median values for the various regions, the envelope (E) gene appeared as the most variable one, showing a median value that was about double compared to the other regions. No significant difference in the heterogeneity of each genomic region was observed when comparing results from the URT and LRT specimens. Detailed patient-specific data for each genome regions are shown in Table S2.

### 3.4. Analysis of Minority Variants in BAL and NPS

**Figure 3:**
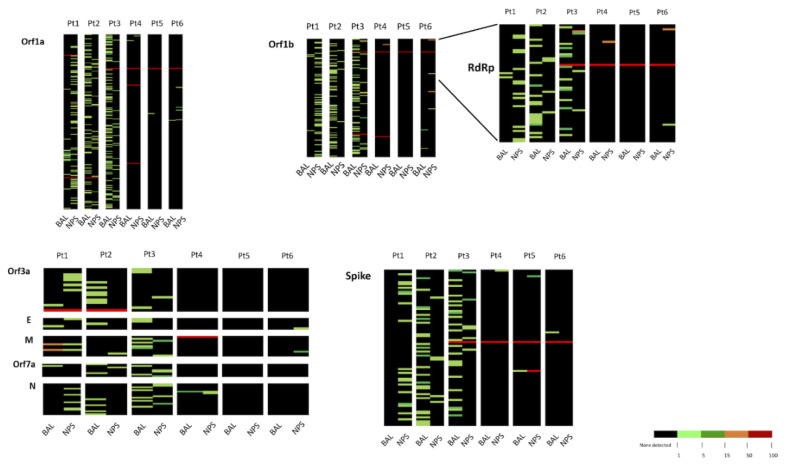
Heatmaps representing intra-host variants in SARS–CoV-2 genome vs. the reference Wuhan-Hu-1 sequence in upper and lower respiratory tract from six Covid-19 patients. Percent of frequency of variants is represented with a color-scale as stated in the figure. For each genomic region individual variants are listed in Table S3.

In the subsequent analysis, we focused our attention on the distribution of variants. In this respect, all the variants present in each sample at a frequency >1% were considered, taking as reference the WuHan-Hu1 strain. Overall, 704 nucleotide variants responding to these criteria were observed (complete list of variants is reported in Table S3). The distribution of variants for each sample is graphically shown as a heat map (Figure 3). Most of the variants were observed with a <5% frequency (minority variants).

Overall, the V clade strains here considered (INMI1, INMI1B, and INMI2) presented a higher number of minority variants than the G clade strains (INMI11, PV-5314, and LO-13075), with the exception of INMI5 (Figure 3 and Table S3). Based on data reported in Figure 3, it appears that BAL and NPS showed a different pattern of variants, which was rather consistent across the different regions for each patient. In some patients, the number of variants was rather high, consistent with the data from Table S2. In addition, in some cases, the number of variants was higher in BAL than in NPS (Pt2, Pt3, Pt4, and Pt5). Finally, no defined pattern of variants was observed in the various patients according to respiratory compartment.

Focusing on the distribution of minority variants in the different gene regions, only 11 substitutions were detected in both lower and upper respiratory district of the same patient: seven in Orf1a, one in Orf1b, two in M gene, and one in N gene. Interestingly, we observed enrichment of a few variants in the M sequence from BAL of Pt1. In a context where other variants remained at the same frequency. In particular, C26681T and G26754T changes were present respectively at 4.6% and 4.2% in NPS; 16.85% and 16.69% in BAL collected at T1; and increased to 87.91% and 88.51% in BAL withdrawn at T2 (Table 3). In addition, in BAL of Pt1, collected eight days after the first sample, SARS-CoV-2 virus strain harbored the A2269T synonymous substitution in orf1a, not detected in the consensus of the other two samples. However, this variant was present only in a minor percentage (10.2%) of sequences of this patient [2].

Two minority variants were observed in S and E protein coding regions of two patients: C21575T non-synonymous mutation (L5F) within the S gene region was present in BAL of Pt3 and in NPS of Pt4 at 11.2% and 1.8%, respectively; and C26251T non-synonymous substitution (S3P) within E gene region was observed in NPS of Pt1 (2.5%) and in BAL of Pt3 (1.3%).

## 4. Discussion

In the present study, we focused on phylogenetic and intra-host variability analysis of the SARS-CoV-2 strains of six patients with severe clinical presentation of COVID-19. A series of clinical specimens from URT as well as LRT were analyzed in order to investigate the presence of possible genetic signatures associated with a lung segregation.

Phylogenetic analysis showed sequence clustering in clade V for the first strains isolated in Italy, and in clade G for strains isolated in later outbreak phases.

In all samples, the presence of viral quasispecies was observed, with variants uniformly distributed along the genome and frequency of minority variants spanning from 1% to ~30%.

For each patient, the patterns of variants in URT and LRT were profoundly different, indicating compartmentalized virus replication, as previously described [12]. However, no clear variant signature for each respiratory compartment was observed, and no significant difference of LRT and URT nucleotide diversity was observed.

Median diversity along the genome was 1.92 (LRT) and 1.24 (URT) nt substitution × 10^−4^/site.

Overall, our analyses confirm the limited variability of the SARS-Cov-2 genome reported so far [13,14,15]. The low diversity highlighted in this study is consistent with previous data, and underlines the need of whole-genome sequence data to perform reliable strain comparison in support of contact tracing.

No clear selection/enrichment pattern has been found in the distribution of minority variants along the whole-genome. Of interest, our analysis revealed that envelope (E) gene is the most variable genomic region of SARS-CoV-2. Clustering of variable sites in the genomic regions of surface protein(s) of several enveloped viruses including Influenza virus, HIV, and HCV has been described [16,17,18]. However, these evidences concern the major receptor-interacting virus protein. E protein plays a central role in virus morphogenesis and assembly, acts as a viroporin, and self-assembles in host membranes forming pentameric protein-lipid pores that allow ion transport. It also plays a role in the induction of apoptosis.

No significant nucleotide differences between LRT and URT samples were detected in the S protein, involved in several different aspects of virus life as receptor binding [19,20,21], tropism [22] and it is also the major target of neutralizing antibodies [23]. We observed two recurrent minority variants in different patients, located in S and E. The C21575T non-synonymous S mutation (L5F) is present in BAL of Pt3 and in NPS of Pt4 at 11.2% and 1.8%, respectively. This change is located in a marginal region of Spike protein, the signal peptide, and is recurrent in different lineages and strains isolated in different countries all over the world, as a result of repeated occasional emergence not followed by fixation; it seems not to have evolutionary advantages [11,24]. The C26251T non-synonymous E substitution (S3P), not previously described, was observed in NPS of Pt1 (2.5%) and in BAL of Pt3 (1.3%). Its significance is not known so far.

Two M gene variants were observed at low frequency in NPS and BAL at T1 and appeared to be enriched in the subsequent BAL sample, suggesting the hypothesis that it could be due to adaptive evolution. M protein is a component of the viral envelope that plays a central role in virus morphogenesis and assembly via its interactions with other viral proteins, so the possible role of M substitutions may be relevant and need further investigation.

## 5. Conclusions

The results of the present study indicate that SARS-CoV-2 displays genetic heterogeneity in respiratory secretions and quasispecies compartmentalization between upper and lower respiratory tract, as previously described for SARS-CoV and MERS-CoV [25,26]. Overall, intra-patient diversity was rather low, and uniformly distributed along the viral genome, although the E protein gene appeared the most variable region. The pattern of minority variants was highly heterogeneous, and no specific district signature could be identified. Due to the low number of patients included in the analysis, these findings may be considered a proof of concept, and more investigation is needed to fix the evidence and identify their pathogenic implications. Nevertheless, the evidence here provided shows that monitoring SARS-CoV-2 genomic signatures is essential in order to gain a better understanding of fundamental host–pathogen interactions and inform drug and vaccine design.

## Figures and Tables

**Table 1:** Demographic, epidemiological, and virological data for the collected patients.

Patient	Pt1B T1	Pt1B T2	Pt1N	Pt2B	Pt2N	Pt3B	Pt3N	Pt4B	Pt4N	Pt5B	Pt5N	Pt6B	Pt6N
**Type of Sample**	BAL T1	BAL T2	NPS	BAL	NPS	BAL	NPS	BAL	NPS	BAL	NPS	BAL	NPS
**Sex**	female			male		male		female		male		male	
**Age**	66			67		53		49		75		54	
**Epidemiological Link, Region (Country)**	Wuhan (China)			Wuhan (China)		Lazio (Italy)		Lazio (Italy)		Lombardia (Italy)		Lombardia (Italy)	
**Date in 2020**	February 4	February 12	February 3	February 3	January 30	March 4	March 4	March 23	March 23	February 21	February 21	March 1	March 1
***C*t**	18.0 *	23.3 *	21.5 *	19.9 *	28.5 *	n.d.	n.d.	14.1 **	16.8 **	n.d.	24.0 *	21.5 *	20.7 *

NPS, nasopharyngeal swabs; BAL, bronchoalveolar lavage; *C*t, cycle threshold for positive signal in E gene-based RT-PCR; n.d., not determined. * Corman et al. 2020 [3]. ** RealStar^®^ SARS-CoV-2 RT-PCR Kit 1.0 assay (Altona Diagnostics, Hamburg, Germany).

**Table 2:** Consensus sequences of study samples: differences vs. Wuhan-Hu-1sequence.

			Pt ID Strain Designation													
Region	nt	Ref *	Pt1B T1 INMI1 BAL1	Pt1B T2 INMI1 AL2	Pt1N INMI1 NPS	Pt2B INMI2 BAL	Pt2N INMI2NPS	Pt3 INMI5BAL	Pt3N INMI5NPS	Pt4B INMI11BAL	Pt4N INMI11NPS	Pt5B PV-5314 BAL	Pt5N PV-5314 NPS	Pt6B LO-13075 BAL	Pt6N LO-13075 NPS	AA Change
5′ UTR	241	C						T	T	T	T	T	T	T	T	-
Orf1ab	2269	A	T		T											Syn
	3037	C						T	T	T	T	T	T	T	T	Syn
	4255	G								T	T					Syn
	10150	T								C	C					Syn
	11083	G	T	T	T	T	T									L3606F
	14408	C						T	T	T	T	T	T	T	T	P4715L
	20268	A						G	G							Syn
	20355	A								G	G					Syn
S	23403	A						G	G	G	G	G	G	G	G	D614G
	24077	G											T			D839Y
Orf3a	26144	G	T	T	T	T	T									G251V
M	26530	A								G	G					D3G
	26681	C		T												Syn
	26754	G		T												G78C

* Nucleotide positions are referred to Wuhan-Hu-1(reference genome MN908947). nt, nucleotide; AA, amino acid; Syn, synonymous substitution; UTR, untranslated region; Orf, open reading fra.

**Table 3:** Nucleotide positions harboring substitution in the membrane protein of respiratory samples from Patient 1.

nt Position	Ref *	Substitution	Pt1N-February 3 (%)	Pt1B T1- February 4 (%)	Pt1B T2- February 12 (%)	AA
**26681**	C	T	**4.63**	**16.85**	**87.91**	Syn
26751	A	G	0.00	0.00	2.30	T77A
**26754**	G	T	**4.20**	**16.69**	**88.51**	G78C
26844	T	C	1.20	0.00	0.00	S108P
27084	G	A	1.50	0.00	0.00	A188T

* Nucleotide positions are referred to Wuhan-Hu-1(reference genome MN908947).

## Supplementary Materials

The following are available online at https://www.mdpi.com/2076-2607/8/9/1302/s1, Table S1: Mean value and range of coverage for SARS-CoV-2 genome of reads obtained by NGS for each analyzed sample. Table S2: Intra-host nucleotide diversity of SARS-CoV-2 genomic regions in BAL and NPS samples of each patient calculated for the whole-genome and for different genes. Table S3: Tables of variants of analyzed samples respect to the Wuhan-Hu-1 reference genome (GenBank Accession No: MN908947). The file is divided into different sheets, each containing variants present along the whole-genome and specific different genes.

## Disclaimer

*The sequences* have been deposited in GISAID and GenBank with accession IDs:

INMI1-B1: EPI_ISL_451298; MT509660
INMI1-B2: EPI_ISL_451299; MT509652
INMI1-N: EPI_ISL_451300; MT509661
INMI2-B: EPI_ISL_451301; MT509653
INMI2-N: EPI_ISL_451302; MT509662
INMI5-B: EPI_ISL_417923; MT509668
INMI5-N: EPI_ISL_451303; MT509654
INMI11-B: EPI_ISL_451304; MT509663
INMI11-N: EPI_ISL_451305; MT509655
PV-5314-B: EPI_ISL_451306; MT509667
PV-5314-N: EPI_ISL_451307; MT509664
LO-13075-B: EPI_ISL_451308; MT509665
LO-13075-N: EPI_ISL_451309; MT509666

## References

[1] WHO (2020). Coronavirus Disease COVID-2019.

[2] Capobianchi M.R., Rueca M., Messina F., Giombini E., Carletti F., Colavita F., Castilletti C., Lalle E., Bordi L., Vairo F. (2020). Molecular characterization of SARS-CoV-2 from the first case of COVID-19 in Italy. Clin. Microbiol. Infect..

[3] Corman V.M., Landt O., Kaiser M., Molenkamp R., Meijer A., Chu D.K., Bleicker T., Brünink S., Schneider J., Schmidt M.L. (2020). Detection of 2019 novel coronavirus (2019-nCoV) by real-time RT-PCR. Eurosurveillance.

[4] Grabherr M.G., Haas B.J., Yassour M., Levin J.Z., Thompson D.A., Amit I., Adiconis X., Fan L., Raychowdhury R., Zeng Q. (2011). Full-length transcriptome assembly from RNA-seq data without a reference genome. Nat. Biotechnol..

[5] Elbe S., Buckland-Merrett G. (2017). Data, disease and diplomacy: GISAID′s innovative contribution to global health. Glob. Chall..

[6] Fu L., Niu B., Zhu Z., Wu S., Li W. (2012). CD-HIT: Accelerated for clustering the next-generation sequencing data. Bioinformatics.

[7] Rambaut A., Holmes E.C., Hill V., OToole A., McCrone J., Ruis C., du Plessis L., Pybus O. (2020). A dynamic nomenclature proposal for SARS-CoV-2 to assist genomic epidemiology. bioRxiv.

[8] Li H., Handsaker B., Wysoker A., Fennell T., Ruan J., Homer N., Marth G., Abecasis G., Durbin R. (2009). The Sequence Alignment/Map format and SAMtools. Bioinformatics.

[9] Bolger A.M., Lohse M., Usadel B. (2014). Trimmomatic: A flexible trimmer for Illumina sequence data. Bioinformatics.

[10] Li H. (2013). Aligning sequence reads, clone sequences and assembly contigs with BWA-MEM. arXiv.

[11] Bartolini B., Rueca M., Gruber C.E.M., Messina F., Carletti F., Giombini E., Lalle E., Bordi L., Matusali G., Colavita F. (2020). SARS-CoV-2 phylogenetic analysis in Lazio region, Italy (February–March 2020). Emerg. Infect. Dis..

[12] Jary A., Leducq V., Malet I., Marot S., Klement-Frutos E., Teyssou E., Soulié C., Abdi B., Wirden M., Pourcher V. (2020). Evolution of viral quasispecies during SARS-CoV-2 infection. Clin. Microbiol. Infect..

[13] Chiara M., Horner D.S., Pesole G. (2020). Comparative genomics suggests limited variability and similar evolutionary patterns between major clades of SARS-Cov-2. bioRxiv.

[14] Shen Z., Xiao Y., Kang L., Ma W., Shi L., Zhang L., Zhou Z., Yang J., Zhong J., Yang D. (2020). Genomic diversity of SARS-CoV-2 in Coronavirus Disease 2019 patients. Clin. Infect. Dis..

[15] Van Dorp L., Acman M., Richard D., Shaw L.P., Ford C.E., Ormond L., Owen C.J., Pang J., Tan C.C., Boshier F.A. (2020). Emergence of genomic diversity and recurrent mutations in SARS-CoV-2. Infect. Genet. Evol..

[16] Bartolini B., Chillemi G., Abbate I., Bruselles A., Rozera G., Castrignanò T., Paoletti; D., Picardi E., Desideri A., Pesole G. (2011). Assembly and characterization of pandemic influenza A H_1_N_1_ genome in nasopharyngeal swabs using high-throughput pyrosequencing. New Microbiol..

[17] Han Q., Jones J.A., Nicely N.I., Reed R.K., Shen X., Mansouri K., Louder M., Trama A.M., Alam S.M., Edwards R.J. (2019). Difficult-to-neutralize global HIV-1 isolates are neutralized by antibodies targeting open envelope conformations. Nat. Commun..

[18] Khera T., Behrendt P., Bankwitz D., Brown R.J., Todt D., Doepke M., Khan A.G., Schulze K., Law J., Logan M. (2019). Functional and immunogenic characterization of diverse HCV glycoprotein E2 variants. J. Hepatol..

[19] Wrapp D., Wang N., Corbett K.S., Goldsmith J.A., Hsieh C.L., Abiona O., Graham B.S., McLellan J.S. (2020). Cryo-EM structure of the 2019-nCoV spike in the prefusion conformation. Science.

[20] Chu H., Chan J.F.W., Yuen T.T.T., Shuai H., Yuan S., Wang Y., Hu B., Yip C.C.Y., Tsang J.O.L., Huang X. (2020). Comparative tropism, replication kinetics, and cell damage profiling of SARS-CoV-2 and SARS-CoV with implications for clinical manifestations, transmissibility, and laboratory studies of COVID-19: An observational study. Lancet Microb..

[21] Chan J.F.W., Kok K.H., Zhu Z., Chu H., To K.K.W., Yuan S., Yuen K.Y. (2020). Genomic characterization of the 2019 novel human-pathogenic coronavirus isolated from a patient with atypical pneumonia after visiting Wuhan. Emerg. Microb. Infect..

[22] Fung T.S., Liu D.X. (2019). Human Coronavirus: Host-Pathogen Interaction. Annu. Rev. Microbiol..

[23] Yu W.-B. (2020). Decoding evolution and transmissions of novel pneumonia coronavirus (SARS-CoV-2) using the whole genomic data. Zool. Res..

[24] Korber B., Fischer W., Gnanakaran S.G., Yoon H., Theiler J., Abfalterer W., Foley B., Giorgi E.E., Bhattacharya T., Parker M.D. (2020). Spike mutation pipeline reveals the emergence of a more transmissible form of SARS-CoV-2. bioRxiv.

[25] Park D., Huh H.J., Kim Y.J., Son D.S., Jeon H.J., Im E.H., Kim J.W., Lee N.Y., Kang E.S., Kang C.I. (2016). Analysis of intrapatient heterogeneity uncovers the microevolution of Middle East respiratory syndrome coronavirus. Mol. Case Stud..

[26] Xu D., Zhang Z., Wang F.S. (2004). SARS-Associated Coronavirus Quasispecies in Individual Patients. N. Engl. J. Med..

